# Exploring the Decision-Making Process of People Living with HIV Enrolled in Antiretroviral Clinical Trials: A Qualitative Study of Decisions Guided by Trust and Emotions

**DOI:** 10.1007/s10728-023-00461-z

**Published:** 2023-07-21

**Authors:** Maria Feijoo-Cid, Antonia Arreciado Marañón, Ariadna Huertas, Amado Rivero-Santana, Carina Cesar, Valeria Fink, María Isabel Fernández-Cano, Omar Sued

**Affiliations:** 1 Department of Nursing, Faculty of Medicine, Universitat Autònoma de Barcelona, Can Domènech, Edifici M, Campus de la Universitat Autònoma de Barcelona, 08193 Bellaterra (Cerdanyola del Vallès), Barcelona, Spain; 2 Grup de REcerca Multidisciplinar en Salut i Societat (GREMSAS), (2017 SGR 917), Barcelona, Spain; 3 Hospital Germans Trias i Pujol, Barcelona, Spain; 4 Canary Islands Foundation-Health Research (FIISC), Tenerife, Spain; 5 Red de Investigación en Servicios de Salud en Enfermedades Crónicas (REDISSEC), Madrid, Spain; 6 Fundación Huésped, Buenos Aires, Argentina

**Keywords:** Antiretroviral, Clinical trial, Decision-making, HIV, Informed consent, Trust

## Abstract

The informed consent is an ethical and legal requirement for potential participants to enroll in a study. There is ample of evidence that understanding consent information and enrollment is challenging for participants in clinical trials. On the other hand, the reasoning process behind decision-making in HIV clinical trials remains mostly unexplored. This study aims to examine the decision-making process of people living with HIV currently participating in antiretroviral clinical trials and their understanding of informed consent. We conducted a qualitative socio-constructivist study using semi-structured interviews. Eleven participants were selected by purposive sampling in Argentina until data saturation was reached. A content analysis was performed. The findings highlight the fact that some participants decided to enroll on the spot, while others made the decision a few days later. In all cases, the decision was based on different aspects of trust (in doctors, in the clinical research site, in the clinical trials system) but also on emotions associated with HIV and/or treatment. Moreover, while people living with HIV felt truly informed after the consent dialogue with a researcher, consent forms were unintelligible and unfriendly. The immediacy of patient decision-making has rarely been described before. Enrollment in an HIV clinical trial is mainly a trust-based decision but this does not contradict the ethical values of autonomy, voluntariness, non-manipulation, and non‐exploitation. Thus, trust is a key issue to be included in reshaping professional practices to ensure the integrity of the informed consent process.

## Introduction

Informed consent is the voluntary agreement of potential participants to enroll in a study, as well as an ethical and legal requirement [22]. The main objectives of the informed consent process are to facilitate good decision-making and obtain valid consent [7]. Informed consent is valid when communication between researchers and potential participants is both effective and does not pre-condition the patient's decision [7, 23]. However, a good decision is one aligned with the patient's values and preferences and based on their understanding of the relevant information for that decision [7].

To obtain informed consent information must be transmitted—through the consent dialogue, including informed consent appointments with recruiters or researchers—and the informed consent form must be signed, including any approved informed consent documentation reviewed by an institutional board [2]. When signing the consent form, participants are supposed to be making an informed, deliberate decision [30] although a de facto signature does not always imply that participants have understood all the information provided nor that they have made a deliberate, rational decision [12]. Deliberate decisions are part of the reasoning theories in which the rational component—understanding the information and risks/benefits assessment—is the main axis upon which the decision hinges. Rational decision-making requires effort and conscious analysis [29].

It is widely recognized that understanding consent information and enrollment can be challenging for participants in clinical trials (CTs) [17]. These challenges have mostly been studied in cancer CTs since cancer is the most common subject of CTs, although a recent literature review identified HIV and cardiovascular disease as the second and third most widely studied diseases [47]. Regarding HIV, issues of CT recruitment and enrollment include barriers and facilitators for people living with HIV (PLWH) in general and also in racial/ethnic minorities [3, 42], adherence to treatment [31], how to increase enrollment [20, 14], and the importance of partners and community in joining CTs [33, 32]. There is evidence showing that in HIV CTs participants do not always understand all the information [44] and that understanding depends on several factors, such as sociocultural norms [34], cultural backgrounds, the health literacy of potential participants, and the written information of both informed consent forms and CT protocols [13].

Although these studies have yielded a substantial amount of knowledge about patients’ limited understanding of informed consent and the enrollment barriers and facilitators among PLWH, the reasoning process behind decision-making in HIV CTs remains mostly unexplored. In particular, several authors have pointed to the need to include patients in the development and execution of clinical studies [1, 18]. The aim of this study is to examine the decision-making process of PLWH currently participating in antiretroviral CTs and their understanding of informed consent. Through a qualitative approach, the results will supplement existing knowledge on decision-making, providing new insight into the process and deepening our understanding of informed consent.

## Data and Methods

### Methodological Approach

A qualitative social constructivist study was conducted [40]. This theoretical approach is based on the need to understand how people construct and interpret social reality in their daily lives, in this case, PLWH participating in CTs. This approach maintains that theories are not discovered but rather that the world we are investigating must be symbolically described through the joint engagement of the interviewee and researcher in the process of constructing realities [11]. Given that we were trying to make sense of the decision-making process of PLWH participating in CTs, the data collection method consisted of face-to-face, semi-structured interviews. The idea behind this data collection technique, and by means of the interaction between the interviewer and interviewee, is that the latter conveys their world to the former, thus providing context to allow for the understanding of their meanings [6].

## Research Context and Participants

The study was conducted at an HIV clinical research site (CRS) in Argentina from November 2016 to February 2017. This CRS is one of the main HIV clinical research sites in Argentina and has participated in several national and international multisite antiretroviral CTs [21]. In Argentina, all citizens have access to the public healthcare system. Additionally, those employed contribute to social health insurance funds: specifically, health insurance is organized and arranged according to each professional union [5]. Most PLWH participating in our study would have received antiretroviral treatment via social health insurance funds or the public health system but antiretroviral drugs tested in CTs are not yet available through these channels. At this CRS, there are various doctors who are also sub-investigators for CTs conducted at the center and who perform medical follow-ups and supervise studies. Each antiretroviral CT at the CRS has different recruitment strategies, but participants arrive through three main channels: (a) associated clinical centers and centers participating in projects with the CRS, (b) a network of infectious disease doctors who are periodically invited to recruit participants, (c) the voluntary counseling program and HIV rapid testing unit at the CRS. Naive participants are recruited basically in two ways: they were diagnosed in either the voluntary counseling program of the CRS or in associated clinical centers. Therefore, some naive participants knew their treating doctor for only one visit and did not know the researcher or care provider before recruitment. When a patient arrives at the CRS for this first appointment, they are interviewed by the CT doctor/sub-investigator, who briefly explains the study and provides the informed consent for them to read. The amount of reading time is based on the length of the consent form. The length of the text is variable as it depends on the specific study protocol (2 to > 30 pages). Therefore, reading time depends on text length and can take up to one hour. After reading the form, time for discussion is allowed to check understanding, resolve doubts and deliberate about alternative options. The patient can have as much time as needed to clarify their doubts, but it usually takes about 45 min to an hour. During the dialogue it is made very clear that even if they do not participate in the CT, they can still use the CRS services or return to their regular care physician. It is emphasized that their HIV care will not be affected whether or not they decide to participate.

For this study, participants were selected by purposive sampling, based on the following criteria: PLWH over the age of 18 participating in any oral antiretroviral CT at the CRS, regardless of whether it was their first time in a CT or they had prior experience. Any other method of drug administration or CT design, such as new prevention or detection strategies, were ruled out because participants might have different experiences. To gather broader perspectives about the topics under discussion, we strived to ensure maximum diversity in terms of sex (men and women), age (25–66), months into the current antiretroviral CT (1–12), education level, and experience with antiretroviral treatment, HIV diagnosis and CT participation. Only two people refused to participate, due to lack of time for interviews. Thus, 11 participants were included in the study (Table 1) and none were financially compensated for their participation.

**Table 1:** Characteristics of participants

Participant	Age	HIV diagnosis	Sexual orientation	Level of education completed	Profession	Months in current antiretroviral CT	PREVIOUS experience before current CT	New HIV diagnosis	Naive or experienced with antiretroviral	Characteristics of antiretroviral CT	Signing the informed consent form
P1_male	52	1998	Homosexual	Trade school	Lawyer	6	Yes	No	Experienced	Phase III multicenter, open-label, randomized study to switch to a new HIV drug	The same day the CT was explained
P2_male	66	2009	Homosexual	Trade school	Retired	5	No	No	Experienced	Pilot, randomized trial to assess RAL + ATV/r* compared with TDF/FTC + ATV/r**	Several days later
P3_male	31	2011	Homosexual	University degree	Teacher	6	Yes	No	Experienced	Phase III multicenter, open-label, randomized study to evaluate a switch to a new HIV drug	Several days later
P4_female	37	2013	Heterosexual	Incomplete secondary school	Housewife	1	No	No	Naive	Phase III, randomized, double-blind, multicenter: evaluating a two-drug regimen compared to the standard of three drugs	The same day the CT was explained
P5_male	44	2014	Homosexual	University degree	Lawyer	8	Yes	No	Experienced	A Phase III multicenter, open-label, randomized study to evaluate a switch to a new HIV drug	The same day the CT was explained
P6_male	28	2015	Homosexual	Secondary school	Cook	12	No	Yes	Naive	Randomized, open-label, phase IV study, designed to compare dual therapy	The same day the CT was explained
P7_male	25	2016	Homosexual	Secondary school	Unemployed	8.5	No	Yes	Naive	Randomized, open-label, phase IV study, designed to compare dual therapy	The same day the CT was explained
P8_male	26	2016	Homosexual	Secondary school	Employed	8	No	Yes	Naive	Randomized, open-label, phase IV study, designed to compare dual therapy	Several days later
P9_male	51	2016	Homosexual	University degree	Self-employed	3	No	Yes	Naive	Randomized, open-label, phase IV study, designed to compare dual therapy	Several days later
P10_male	41	2016	Heterosexual	Secondary school	Musician	6	No	Yes	Naive	Randomized, open-label, phase IV study, designed to compare dual therapy	Several days later
P11_male	34	2016	Homosexual	University degree	Math teacher	2	No	Yes	Naive	Phase III, randomized, double-blind, multicenter: evaluating a two-drug regimen compared with the standard of three-drugs	Several days later

*RAL + ATV/r: Raltegravir + Ritonavir boosted Atazanavir

**TDF/FTC + ATV/r: Tenofovir/Emtricitabina + Ritonavir boosted Atazanavir

### Data Collection

The first author explained the project and inclusion criteria to recruiters at the CRS. Recruitment was performed in person by doctors during CT follow-ups or by the interviewer (MF), either in person or by phone. MF was there for a six-month postdoctoral stage and had not met study participants beforehand. Semi-structured face-to-face interviews were conducted by MF, who is trained in qualitative methods. The interview followed a script to explore the participant’s decision-making process and understanding of the informed consent.

The script consisted of three main issues: experiences participating in antiretroviral CTs, how decisions were made, and informed consent (dialogue and forms). It began by contextualizing the interview and the CT in which the interviewee was participating so the group would feel at ease and then went deeper into the study topics. Some of the questions asked to guide the interviews were: “How would you describe your experience of participating in antiretroviral CTs? Could you explain what you know about the CT you are participating in? Why did you agree to participate in a CT? What information did you receive when you were invited to participate in a CT and what did you discuss with your doctors? How would you define informed consent? What do you expect the end of the trial to be like? Could you tell me about your rights as a participant in a CT? Could you tell me which is the most important for you?” The first draft of the script was reviewed by one PLWH who had experience with antiretroviral CTs and by one health professional working in CTs. The interviews lasted from 35 min to 1 h 40 min and were audio-recorded and transcribed verbatim. The interviews were conducted until data saturation was reached, which was based on repeated responses within the interview of each participant [43].

### Data Analysis

A content analysis was performed manually by two authors (MF, AH). They first coded the interviews independently and then jointly to build the framework of the initial codes. They then worked independently to sort codes into different categories and subcategories by comparing differences and similarities. Lastly, working together, they identified final categories and abstracted them into main categories (Table 2), resolving any differences by consensus. Additionally, two expert researchers (AR, AA), who did not participate until the core team reached consensus about findings, reviewed both the coding process and the findings.

**Table 2:** Overview of the coding process

Theme	Decision management: deciding on the spot or days later				
Categories	Multiplicity of trust			Emotions	
Subcategories	Trust in doctors	Trust in the institution	Trust in the CT system	Associated with HIV	Associated with ART
Codes	Feeling emotionally supported and comfortable in consent dialogue Humanized care	Feeling emotionally supported Humanized care Good reputation (both nationally and scientifically) vs bad reputation of the public health and social health insurance funds systems	Expectation of receiving extra/consistent care Full availability of the physician/researcher Management efficacy in monitoring Antiretroviral clinical trial regimens are less toxic than regular care ones	Feeling vulnerable due to being recently diagnosed with HIV Perception of being a guinea pig Fear of progression to AIDS	There is no choice: you need treatment Side-effect fatigue of previous antiretroviral treatment Fear of adverse effects of CT treatment Not giving in to fear of adverse effects

Theme	The importance of personal communication in informed consent			
Categories	Explaining the informed consent: the human factor	Unfriendly informed consent forms		
Subcategories	Understandable consent dialogue	Unintelligible text	Frightening adverse effects	Rights & duties
Codes	Clear and detailed information The guinea pig metaphor Resolving doubts Complete dialogue with physicians	Scientific writing, not easy to read Not adapted to different education levels	Too long, too many pages Too much information about adverse effects	Always focused on bad news Who is responsible for the transition when leaving the CT

### Ethical Considerations

The Research Ethics Committee of Fundación Huésped approved the study (Study Fh-23). Individuals participated voluntarily, signing the informed consent document after receiving thorough information about the study and being assured of their right to withdraw at any time. Written informed consent explicitly included the possibility of the results being published. This was also explained in the consent dialogue. Participants were reassured about data anonymity and confidentiality.

### Rigor

The strategies used to maintain the credibility of the study included an interview guide to ensure coverage of important research topics. For the sake of credibility, participants were sent the transcriptions of their interviews and asked to modify anything that did not accurately reflect their discourse. To avoid recall or social desirability bias, they were asked not to change the content, just the wording. Two researchers with experience in decision-making and qualitative methods conducted an independent analysis to reinforce confirmability. And to better ensure the accuracy of the results, a first draft of the paper was submitted to one participant for validation. Dependability was strengthened by the accuracy and description of data collection. Analysis and participants’ quotations illustrating the main themes contributed to the consistency of findings.

## Findings

All participants, aged 25 to 66, were enrolled at the time in a phase III or IV antiretroviral CT. We categorized our findings around two main subjects: “Decision management: deciding on the spot or days later” and “The importance of personal communication in informed consent”.

### Decision Management: Deciding on the Spot or Days Later

Five people decided on the spot, immediately after the informed consent explanation, while six people decided several days later. The issues involved in postponing the decision or not were common and simultaneously influenced the decision, just to different degrees. They are grouped into *Multiplicity of trust* and *Emotions* associated with HIV and/or antiretroviral treatment.

#### Multiplicity of Trust

Trust guided both types of decision: granting consent on the spot or doing so later. But trust was key in multiple directions: trust in the doctor and institution for people who had been living with HIV for some time, and trust in the CT system—i.e., access to lifesaving ART—for those who were newly diagnosed. Most participants decided to participate because they trusted the physicians who informed them in the consent dialogue. The value associated with health professionals was not just their clinical expertise but the humanized care they offered: newly diagnosed people who experienced emotions such as fear of HIV progressing to AIDS felt the support of the professionals in coping with the diagnosis.

I felt emotionally supported [by doctors], and comfortable, and I needed that. When you have been recently diagnosed with HIV and you receive all of that [emotional support and information] … that is what you need to decide (P6).

Treatment-experienced people also valued this human quality in doctors and sometimes associated it with a decrease in the feeling of being a "guinea pig". For those newly diagnosed, this feeling was also mentioned but dissipated as a trustworthy relationship with the practitioner developed.

It is related to feeling treated like a human being. When one [professional] shows a humanized attitude towards PLWH, it diminishes this sensation, it diminishes that guinea pig feeling (P3).

[Guinea pig feeling] It starts to dissipate when you understand that they are not playing with you, they are helping you because they are trying to make a change (P4).

Participants also trusted the institution. The CRS has a good reputation in Argentina, and most participants had a poor opinion of the public health and social health insurance funds systems because of what they consider dehumanized care.

(…) the staff who work there [public hospital] aren’t prepared (…) They told me I was seropositive. Such a blow to deal with. I said, “What did you say?” (P7).

(…) with the FH reputation, if they decided to try a new medicine with me, I would do it (P1).

Trust in the CT system was also important. In this case, trust was oriented mainly towards the expectation of receiving higher quality care by participating in the CT. Participants expected better and longer follow-up and closer support from the doctor if they consented. Doctors' support was not limited to the consent dialogue but included them staying by the patient’s side throughout the trial. This trust in the CT system also indicates that they would have access to a better and more tolerable ART. In fact, for people recently diagnosed participating in the CT was access to lifesaving ART.

Before I used to take five pills, that is, two medications (...) With this one I take only one, just one, and I had no changes (...) I am happy because it is much simpler with one.... I always felt good (P3).

Trust in the CT system was also based on its efficacy. At the CRS, the prescription of medication and clinical follow-up are guaranteed within a single CT appointment while in the public system patients needed at least two appointments to get standard treatment. Some participants with experience in antiretroviral CTs wanted to sort out the overbearing bureaucracy required to obtain antiretroviral treatment in the public system.

I needed a break from the social health insurance funds [system]. Adverse effects do not fuck me up, what does fuck me up is bureaucracy, wasting my time in bureaucracy and queuing, waiting for everything, when everything could be easily done in one day (…) social health insurance funds are a disaster (P3).

#### Emotions

People recently diagnosed with HIV had different conflicting emotions, including fear of HIV progressing to AIDS, fear of the HIV diagnosis itself, and uncertainty about their future living with HIV. Such feelings either encouraged or constrained their perception of antiretroviral treatment and their ability to assess whether one medication was better than another. However, the core emotion related to treatment, which was also common among people with experience with treatment, was that they felt they had no choice: they needed treatment to control the virus, and not taking treatment was not an option, so they considered the trial an opportunity to get it.

When you are diagnosed with HIV, and they tell you: you need ART, there is no decision to make… you go into the trial (P6).

At the time of diagnosis, you are in a situation of vulnerability because any treatment offered to you will seem good due to fear… a lot of fear about what was going to happen to me… I have a big mambo in my mind, I couldn't understand it at all, I had HIV overnight. Now, after 6 years [it] is totally different (P3).

Although they felt vulnerable, they never expressed that doctors or CRS were doing something wrong or taking advantage of them. The ignorance and fear mentioned here were related to uncertainty about their future living with HIV, fear of the HIV diagnosis itself, and fear of HIV progressing to AIDS.

Fear of adverse effects of CT treatment and side-effect fatigue was also important for those who decided on the spot, whether they were experienced or naive participants. They all wished to avoid antiretroviral treatment toxicity. Those with experience with trials or antiretroviral treatment wanted to get off their current antiretroviral treatment regimen, while those new to trials were hoping for combinations with less toxicity following the information received in the consent dialogue process.

I changed [and joined the CT] for two reasons, and I’m going to confess: my triglyceride levels had been triggered and I had sexual dysfunction due to ritonavir (P5).

I thought that my body would not be able to hold up, to deal with side effects (P4).

Participants who made their decision a few days later made it on their own or with the support of their social network (family, partners, friends). They were also afraid of side effects and becoming fatigued by the adverse effects of CT treatment. Management of this fear was different: some decided to stop reading the informed consent forms while others did not want fear to rule their lives and decided not to give in to it.

I read everything at home, calmly… at the beginning, I was afraid of the large number of adverse effects, but later I said to myself: you do not have to live with fear (P11).

I read as much as I could, and I checked it with my doctor and my friend (P9).

### The Importance of Human Communication in Informed Consent

From the participants’ perspective, while the consent dialogue was understandable, the informed consent forms were not easy to comprehend and the right to leave a trial was a real, and sometimes unresolved, concern. We divided this section in two main categories: *Explaining informed consent: the human factor* and* Unfriendly informed consent forms.*

#### Explaining Informed Consent: The Human Factor

Our participants considered the consent dialogue a complete dialogue with their physicians because they received all the necessary information to decide about enrollment. Through that conversation, they found that everything became clear, they resolved doubts about CTs, side effects and about the metaphor of being “guinea pigs.” They also valued personalized attention, humanized care and the time spent resolving doubts.

My doctor told me exactly what the CT protocol was going to be like and gave me a bunch of papers to read and decide whether or not to participate. He told me I would receive the medication for two years and I would go through a lot of analysis (P10).

She [the physician] told me that I was not a guinea pig (…) (P7).

Although they described the consent dialogue as helpful and clear, when asked how they resolved their doubts about the written information, some participants seemed to hesitate about their previous assessment of the interaction. When asked what would have helped them resolve their doubts, they suggested that their physician should have talked more about their doubts regarding side effects. Some participants pointed out that physicians should decode technical or scientific words in the informed consent form.

A good professional (…) should be able to communicate well and decode all scientific or technical words into a language the patient would understand (P5).

#### Unfriendly Informed Consent Forms

For most participants, some of the information in the informed consent form was difficult to understand. These documents used too many scientific words that made them hard to read and understand, with adverse effects as the highest concern. Moreover, there was much more information that frightened rather than reassured them. Most participants would emphasize the possibility of not experiencing adverse effects in the informed consent forms so that the benefits could be better explained (which would encourage participation).

When you see all the adverse effects, it’s horrible, you think that taking this medication is suicide (…) A more positive perspective is necessary to focus on the beautiful part of participation (P9).

They could use much easier to read language, I mean, without being colloquial, it could be more understandable (P5).

Many participants were not aware of the duties and rights mentioned in the informed consent form. Although all rights and duties needed a clearer explanation, the right to leave the trial raised many doubts because there was no information about the different aspects of this transition: what would be the new drug combination or who would oversee the transition to standard care. This was a real concern for study participants, but the informed consent form only explained the right to leave the trial, not the details of the transition or who would be responsible for it, either when interrupting the trial or at the planned end of it. The uncertainty of transitioning (following CTs) was associated with distrusting institutions that provided standard care because study participants were seeking high-quality health care, the same as that received while participating in the ART-CT.

You have the right to leave but then what will happen? You must start again […] What happens to me? This is not answered in the informed consent sheet (…) Where do I go? Do I stay here [at the institution]? (P9).

## Discussion

This study highlights the fact that some participants decided to enroll on the spot, while others made the decision a few days later. In all cases, the decision was based on different aspects of trust (in doctors, in the CRS, in the CT system) but also on emotions associated with HIV and/or treatment. Moreover, while PLWH felt truly informed after the consent dialogue with a researcher, consent forms were unintelligible and unfriendly.

It is assumed that informed consent is valid when the potential participants receive sufficient relevant information, adequately understand this information, and decide without coercion or influence. Considering our results, a threefold debate arises as to the validity of the decisions made: a) whether decisions based on a multiplicity of trust (in the doctor, CT system, etc.) and emotions are ethically valid or not, since preferences and values are prioritized; b) whether deciding on the spot is consistent with the rational or deliberative model of decision-making; c) whether differences in the understanding of the information provided in the consent dialogue and the written information determine the validity of the decision, given that there is no structured protocol for the consent dialogue that ensures enough information is provided.

### Decisions Based on a Multiplicity of Trust and Emotions

Regarding the first debate—trust and emotion-based decision—several studies have identified the health professional as potentially the most challenging variable to influence patients' participation in CTs (for cancer, HIV and cardiovascular disease, surgery) [12, 47, 28, 25,35]. In Western countries [9], patients usually trust healthcare professionals but distrust the healthcare system [15]. That was the case in our results, where PLWH distrust the public system but there is a general atmosphere of social confidence in the CRS. Trust in the CT system was also important in the PLWH in our study. Access to unavailable or privately funded drugs or perceived benefits of participating had a positive impact on patient enrollment [4]. Regarding emotion-based decisions, a cancer RCT setting showed that the decision to participate was more emotionally driven than consciously deliberated [16]. When comparing the cognitive processing strategies used by cancer patients and the general population to decide whether to enroll in a potential trial, emotions played a substantive role in the cancer patients' decision-making while cognitive processing strategies guided the decisions of the general population [51]. Evidence reveals that when facing situations that pose a serious life threat (such as being diagnosed with cancer or HIV), there are many difficulties in cognitively processing information, and decisions are often made based on emotions [16].

A recent study noted that under appropriate circumstances, trust-based consent was not morally inferior to informed consent [28]. In other words, the essential moral values that validate informed consent (autonomy, voluntariness, non‐manipulation, and non‐exploitation) may be present in both information-based and trust-based decisions. Various authors suggest that the values of would-be participants that influence their decisions may be considered just as valid as the value of rationality stemming from understanding the information and deciding by weighing pros and cons [31, 27]. Therefore, the fact that the values of the patient (such as trust or different emotions) take precedence in the decision-making does not invalidate the informed consent process. However, it should be noted that, from the perspective of relational autonomy, this trust in the professional should be questioned upfront given the power hierarchies inherent to the physician/sub-investigator/potential participant relationship [4]. These power differences and the inherent nature of the patient/doctor relationship raise questions about whether patients might feel socially limited or pressured to participate in research [4].

### Deciding on the Spot and the Deliberative Model of Decision-Making

Regarding the second debate, it should be noted that the immediateness of patient decision-making has rarely been described before and the authors found no evidence to date in antiretroviral CTs but did find some in cancer trials. In one study, the decision to participate in CTs was immediate in one-third of participants while it took some time for another third [49]. In another study, the decision was obvious or immediate when patients were invited to a CT adapted to their type of cancer [16]. Immediate decisions are usually associated with intuitive decision-making and, at times, guided by emotions [16]. One paradigm of cognitive psychology advocates two different ways of going through the decision-making process: a slow, cognitive, self-deliberative and conscious way (mentioned above as rational decision-making), and a fast, automatic and unconscious way [19]. While the former relies on deliberation, the latter relies on experience and emotions. Some authors refer to it as the heuristic-systematic model [19]. Heuristics is a set of cognitive tools that people use to quickly solve problems with limited information in complex environments, and it is not a less effective version of rationality. We cannot state whether on-the-spot decision-making in our results was deliberative or not when one study indicates that the minimum time required for a person to make a deliberate decision and sign the consent form is four days [36]. When discussing the immediacy of the decision to participate, other authors suggest that written information may be less important than previously considered [16], and even that some participants have already made their decision before the consent dialogue, which supports the assumption that understanding the consent form or consent dialogue may not be important to them [27, 36]. However, some authors consider immediate decisions equally valid for informed consent [16] because they respond to one of the possible ways human beings make decisions, according to the theories of dual processing in decision-making [16].

### The Importance of Personal Communication on Informed Consent

Regarding the third debate, our results suggest that the consent dialogue was more understandable than the informed consent form. Recently published evidence points out that one-to-one discussions with study participants is most effective in improving their understanding of the informed consent [39], given that written information has less impact on their decision than a verbal exchange [16, 24]. In participant-led appointments, participants express their concerns, and it is easier to check what have they understood, although not all necessary information is offered [46]. Moreover, it is unusual to ask would-be participants for feedback about the informed consent process [10]. It remains an aspect to be improved since the proportion of participants who understand the informed consent process has not increased over the last 30 years [45]. While the content and presentation of informed consent form are well regulated [46], there is no supervision of the amount of oral information or the way it is expressed in the consent dialogue, so its effects on participants' understanding [8], as well as the possible use of coercion [50], remain unknown. There is still some tension between what potential participants need to decide and what is legally and ethically required, even though both the content and presentation of the informed consent form are highly standardized [46]. However, a recent study shows that patients do not specifically emphasize key aspects as rights despite these being defined as priority areas when making an informed decision [26]. Therefore, two questions come to mind: how detailed does information need to be in the consent dialogue for a person to sign the informed consent? And, when and how it should be provided [46]?

Our results demonstrated that PLWH wanted more detailed information about the right to withdraw from the trial at any moment and transition to standard HIV care. The transition to regular care raised many doubts. The evidence clearly recommends describing post-trial services in CT protocols and the informed consent form or offering additional assistance to help trial participants express their ambivalence or doubts about the challenges of a future transition to routine care [13, 52]. Although current ethical guidelines propose obligations of CT researchers, such as the need to ensure continued access to necessary HIV treatments, psychosocial support and other services [37], PLWH are scared and worried when the transition to regular HIV care nears [37, 41]. Evidence points to psychological stress related to loss of quality care and loss of material benefits, concerns about how to access care after exiting the trial, difficulties associated with linkage to care facilities, and difficulties in coping with treatment and transportation costs [37, 38]. CTs offer participants many advantages that are not found at the regular HIV care centers [41].

This study has some limitations. This is a small sample from a specific setting. However, it provides a detailed description of the population, and it is one of the main clinical research sites for HIV in Argentina. Thus, the study provides credible, reliable results and stands out as one of the few examples in the literature on the reasoning process of decision-making in HIV CTs. The results are presented as a first step towards future in-depth research. Other limitations include the fact that the purposive sampling was guided by very specific criteria (being part of an ongoing trial and having made the decision in the past). Additionally, involving doctors in the qualitative recruitment process likely conditioned the response of the participants; as shown in the findings, participants trusted their doctors. Although we acknowledge that both limitations might lead to a selection bias, our results shed light on an area of HIV healthcare where evidence is scarce and raise new issues that deserve further investigation, all of which can be considered a strength. Interviewing participants an average of six months after making the decision rather than while they are in the process may also have induced a recall bias. To circumvent this bias, we suggest that future studies focus on exploring decision-making at the consent dialogue session. Moreover, the opinion of patients who did not grant consent was not included in this study as it was not possible to access them. Lastly, we only explored the perspective of patients. Comparison with patients who refused to participate and an evaluation of healthcare provider perspectives would be necessary to better understand the dynamics of this decision-making process in context. Nonetheless, the contribution of this study is the multiplicity of trust- and emotion-based influences and the value of dialogue to clear up the elusiveness of a consent form.

## Conclusions

Our findings suggest that decisions to enroll in an antiretroviral CT are trust-based and emotionally guided. All potential participants made their decision either on the spot or a few days later. Doctors play an important role because they often provide information about CTs and create a climate of trust in which patients feel comfortable agreeing to participate in research. Trust-based and emotionally guided decisions do not invalidate informed consent as they do not contradict per se the ethical values of autonomy, voluntariness, non-manipulation, and non‐exploitation. But from a relational autonomy approach, trust-based decisions could be compromised.

Given this scenario, the integrity of the staff or, at least, the integrity of the consent process could be ensured by a) training professionals on how to obtain informed consent—staff members report that they learned by doing and not through training, b) including an observer to monitor recruitment and ensure that no type of coercion takes place during the process. To ensure that all the relevant information is provided in the consent dialogue it is necessary to: a) develop a standard script to guide the discussion/conversation around all the essential issues in the consent process; b) assess the understanding of the consent process and measure whether people who have consented are, or feel, fully informed and are deciding freely. Transition to HIV regular care could also be mitigated using patient navigators, as has previously been proven effective.

## Data Availability

The data that support the findings of this study are available on request from the corresponding author [AAM]. The data are not publicly available due to containing information that could compromise research participant privacy/consent.
